# Impact of Short and Long Interpregnancy Intervals on Neonatal Outcomes: A Multiclassification Cohort Analysis

**DOI:** 10.3390/healthcare14070826

**Published:** 2026-03-24

**Authors:** Gizem Boz Izceyhan, Resul Karakuş, Mina Erbıyık

**Affiliations:** Department of Obstetrics and Gynecology, Zeynep Kamil Women and Children’s Diseases Training and Research Hospital, Istanbul 34668, Turkey; resul-karakus@hotmail.com (R.K.); drminaerbiyik@gmail.com (M.E.)

**Keywords:** interpregnancy interval, birth spacing, neonatal outcomes

## Abstract

**Introduction:** Interpregnancy interval (IPI) plays a critical role in neonatal health, yet optimal spacing remains controversial. This study assessed neonatal outcomes across short and long IPI using three complementary classification approaches to identify consistent patterns of risk. **Materials and Methods:** In this retrospective cohort study, medical records of 1194 women with a prior live birth who delivered singleton pregnancies in 2024 at a tertiary referral center were analyzed. IPI was calculated as the delivery-to-conception interval (LMP + 14 days). Three IPI classification systems were applied: (1) classical cut-offs (<6, 6–11, 12–23, 24–59, and ≥60 months), (2) quartiles, and (3) tertiles. Primary outcomes included preterm birth, low birth weight (LBW), and NICU admission. Multivariable logistic regression models adjusted for maternal age, gravidity, and previous cesarean delivery. **Results:** Short IPI (6–11 months) demonstrated the highest NICU admission rates (29.4%). Very long IPI (≥60 months) showed the highest prevalence of LBW (16.6%). Multivariable regression analysis revealed that intervals ≥ 24 months were independently protective against preterm birth (24–59 months: aOR 0.48, *p* = 0.002; ≥60 months: aOR 0.58, *p* = 0.042), while maternal age increased preterm birth risk by 7% per year. Short IPI (6–11 months) and very long IPI (≥60 months) independently increased NICU admission risk (aOR 2.29, *p* = 0.002 and aOR 1.61, *p* = 0.036, respectively). Previous cesarean delivery was an independent predictor of NICU admission (aOR 1.35; *p* = 0.048). **Conclusions:** Short and very long IPIs are associated with increased neonatal morbidity, particularly NICU admission, while the apparent preterm risk in long intervals is largely mediated by maternal age. Once adjusted, IPIs exceeding 24 months demonstrate protective effects against preterm birth. However, the rising trend toward LBW and NICU admission in intervals beyond 5 years suggests that birth-spacing counseling targeting an optimal window of 18–24 months provides the best balance in minimizing competing neonatal risks.

## 1. Introduction

Interpregnancy interval (IPI)—defined as the time between a previous live birth and conception of the subsequent pregnancy—is increasingly recognized as a critical and modifiable determinant of neonatal health [1]. The World Health Organization (WHO) recommends waiting at least 24 months after a live birth before attempting the next pregnancy to reduce maternal and neonatal morbidity [2]. Despite these global recommendations, substantial heterogeneity persists in defining “short” and “long” IPI, with thresholds ranging from <3 months to <24 months for short intervals [3,4]. This variation complicates direct comparison between studies and contributes to inconsistent clinical guidance.

Professional societies emphasize the clinical consequences of inadequate spacing. The American College of Obstetricians and Gynecologists (ACOG) highlights increased risks associated with very short intervals, advises avoiding conception within 6 months, and recommends individualized counseling for women considering pregnancy before 18 months [5]. In their joint consensus on interpregnancy care, the Society for Maternal–Fetal Medicine (SMFM) and ACOG identified IPI as a pivotal period for improving not only neonatal outcomes but also long-term maternal health [6]. This consensus underscores that interpregnancy care should integrate reproductive planning, chronic disease management, psychosocial support, and optimization of maternal health before subsequent conception.

Multiple observational studies and meta-analyses have demonstrated that short IPI (<18–24 months) is associated with increased risks of preterm birth, low birth weight (LBW), and perinatal mortality [7,8]. A large prospective cohort study similarly showed that pregnancies conceived within 18 months carry 35% higher risk of preterm birth and 120% higher risk of LBW [9]. Conversely, evidence suggests that excessively long intervals (>60 months) may also pose risks, potentially reflecting a loss of physiological adaptation or increased maternal comorbidities accumulated during the extended interval. A nationwide Swedish cohort study demonstrated increased neonatal morbidity among women with long IPI, supporting the hypothesis that both extremes of spacing may be detrimental [10].

In addition to definitional inconsistencies, methodological differences in how IPI is calculated—birth-to-birth, delivery-to-conception, or inter-delivery interval—further challenge standardization. The WHO suggests that in low- and middle-income countries, the inter-delivery interval may better reflect maternal depletion and recovery patterns [11]. This variation underscores the need for systematic analyses using multiple classification approaches within the same population.

Interpregnancy intervals are influenced by a complex interaction of biological, sociocultural, and economic determinants [7]. Factors such as breastfeeding practices, postpartum amenorrhea, access to effective contraception, maternal educational status, and cultural norms regarding family size can substantially influence birth spacing decisions. Socioeconomic conditions and access to healthcare services may further affect reproductive planning and the timing of subsequent pregnancies. Understanding these determinants is important for interpreting observed associations between birth spacing and neonatal outcomes [12].

Given these uncertainties, the present study aimed to evaluate neonatal outcomes across the IPI spectrum using three different classification methods: classical cut-offs, quartiles, and tertiles. This multipronged approach allows the identification of consistent risk patterns while also revealing potential threshold effects not captured by traditional definitions. We hypothesized that both short and long IPIs would be associated with an increased risk of preterm birth, LBW, and NICU admission. By analyzing a large, contemporary cohort from a tertiary referral center, this study seeks to contribute clinically actionable evidence to guide postpartum counseling and optimize birth spacing recommendations.

In the context of Turkey, and particularly in a metropolitan hub like Istanbul, IPIs are influenced by a unique intersection of modern healthcare access and evolving social norms. While the Turkish national healthcare system provides broad access to family planning services, the actual utilization of effective contraception often varies according to maternal educational status and regional cultural preferences regarding family size. Furthermore, the rapid urbanization of Istanbul has led to a dual demographic trend: a significant portion of the population continues with traditional short spacing, while another segment, often due to economic participation and delayed marriage, exhibits the longer intervals that our study identifies as increasingly common.

## 2. Materials and Methods

### 2.1. Study Design and Setting

This retrospective cohort study was conducted at the Zeynep Kamil Women and Children’s Diseases Training and Research Hospital, a high-volume tertiary referral center in Istanbul, Türkiye. Medical records of women who delivered between 1 January and 31 December 2024 were reviewed. Clinical data were retrieved from the hospital’s electronic medical record system. Data extraction was performed manually by trained investigators to ensure accuracy and completeness of obstetric and neonatal variables. This study included women with at least one prior live birth and a singleton pregnancy during the study period. Exclusion criteria were: (1) multiple gestations, as plurality is a strong independent risk factor for preterm birth and low birth weight that could confound the analysis of IPI-related outcomes; (2) major congenital anomalies; (3) missing or unreliable obstetric data; and (4) pregnancies ending before 24 weeks of gestation. A total of 1500 women were screened, and after exclusions, 1194 were included in the final analysis.

**Figure 1:**
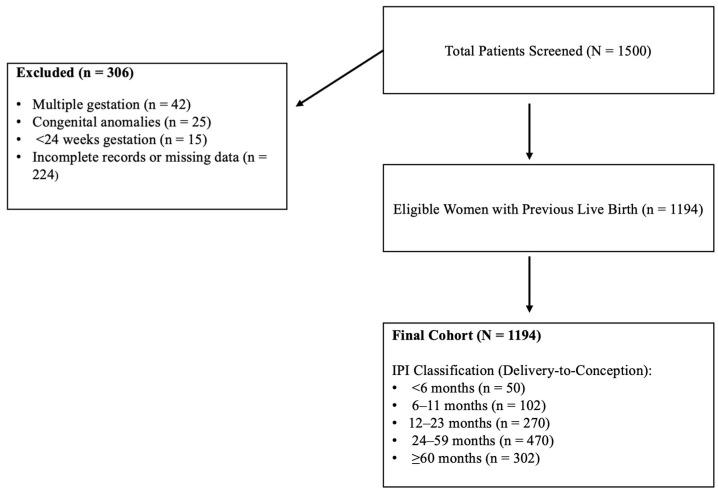
Flow diagram of participant inclusion, exclusion, and final study population.

A flow diagram of participant selection and exclusions is presented in Figure 1.

### 2.2. Definition and Calculation of Interpregnancy Interval (IPI)

IPI was defined as the delivery-to-conception interval, estimated by adding 14 days to the last menstrual period (LMP) of the index pregnancy, a method commonly used in epidemiological studies evaluating birth spacing and perinatal outcomes [12]. When available, first-trimester ultrasound dating was used to confirm gestational age and correct discrepancies in LMP-based estimates. The final IPI calculation represented the delivery-to-conception in completed months.

Because there is no universally accepted IPI threshold, we categorized IPI using three complementary methods:

1. Classical (Literature-Based) Categories:
  - <6 months;
  - 6–11 months;
  - 12–23 months;
  - 24–59 months;
  - ≥60 months.
2. Quartile-Based Categories:
  - Q1 ≤ 17.8 months;
  - Q2 17.9–32.9 months;
  - Q3 33.0–60.2 months;
  - Q4 ≥ 60.3 months.
3. Tertile-Based Categories:
  - T1 ≤ 22.7 months;
  - T2 22.8–48.6 months;
  - T3 ≥ 48.7 months.

This multimethod approach allowed a more nuanced assessment of potential threshold effects.

### 2.3. Maternal and Neonatal Variables

Maternal variables:

- Age;
- Gravidity and parity;
- Previous cesarean delivery;
- Gestational age at delivery.

Neonatal outcomes:

- Preterm birth (<37 weeks);
- Early preterm (<34 weeks);
- LBW (<2500 g);
- Macrosomia (≥4000 g);
- 1- and 5-min Apgar scores;
- NICU admission.

*NICU Admission Criteria*

To minimize misclassification, NICU admission was defined using institutional standardized criteria:

- Respiratory distress requiring oxygen supplementation > 2 h;
- Prematurity < 34 weeks;
- Hypoglycemia < 40 mg/dL requiring IV therapy;
- Suspected sepsis requiring evaluation;
- Need for CPAP or mechanical ventilation;
- Any condition requiring continuous bedside monitoring.

NICU admission criteria were standardized; however, we acknowledge that admission thresholds may reflect institutional practice patterns. The primary indications for admission were respiratory distress requiring oxygen supplementation and prematurity-related complications.

These criteria ensured uniform assessment across all IPI categories.

### 2.4. Handling of Missing Data

Missing data were minimal (<3%) and were handled via complete-case analysis. Sensitivity analyses excluding all participants with missing covariates yielded consistent findings.

### 2.5. Statistical Analysis

Continuous variables were tested for normality using the Shapiro–Wilk test. Nonparametric variables were compared using the Kruskal–Wallis test; categorical variables were compared using chi-square or Fisher’s exact tests.

Both crude (unadjusted) and adjusted odds ratios (ORs) with 95% confidence intervals (CIs) were calculated to demonstrate the effect of potential confounders. Separate regression models were performed for preterm birth, NICU admission, and low birth weight (LBW).

Covariates (maternal age, gravidity, and previous cesarean delivery) were selected based on biological plausibility and prior evidence of confounding. Multicollinearity was assessed using variance inflation factors (VIF < 2). Model calibration was evaluated using the Hosmer–Lemeshow goodness-of-fit test.

### 2.6. Power Analysis

Based on expected baseline rates of preterm birth (10–15%) and an anticipated OR of 1.8 for short IPI, the available sample size (*n* = 1194) provided >80% power at α = 0.05 to detect clinically meaningful differences.

Statistical analyses were performed using SPSS v25.0 (IBM Corp., Armonk, NY, USA). A two-sided *p* < 0.05 was considered significant.

## 3. Results

### 3.1. Study Population and IPI Distribution

The final cohort included 1194 women. Baseline maternal characteristics across classical interpregnancy interval categories are presented in Table 1 to allow assessment of group comparability. The mean IPI was 44.6 ± 38.7 months, with a median of 32.6 months (range: 1.9–322.8). Q1 and Q3 thresholds were 17.8 and 60.2 months, respectively. Using classical cut-offs, the largest proportion of women (64.7%) had IPI ≥ 24 months, while only 4.3% had IPI < 6 months (Table 2: Classical IPI distribution).

### 3.2. Maternal Characteristics

Across all classification methods, maternal age and gravidity increased progressively with longer IPI. Women with IPI <12 months were younger and had lower gravidity/parity, while those with IPI ≥24 months were older with higher gravidity.

In classical groups:

- Maternal age ranged from 24.8 years (<6 months) to 33.5 years (≥60 months).
- Gravidity and parity followed similar trends.
- Previous cesarean delivery was highest in the ≥24-month group (*p* = 0.04).

(Table 3)

****

Neonatal Outcomes (Classical Categories).

**Table 3:** Maternal and neonatal findings by IPI groups.

IPI Category	*N*	Mean Maternal Age	Preterm Birth (%)	LBW (%)	NICU (%)
<6 months	50	24.8	4.0%	0.0%	16.0%
6–11 months	102	26.2	9.8%	9.8%	29.4%
12–23 months (ref.)	270	27.6	15.6%	11.9%	16.3%
24–59 months	470	30.2	9.4%	9.8%	21.3%
≥60 months	302	33.5	13.9%	16.6%	24.5%

Abbreviations: IPI, interpregnancy interval; LBW, low birth weight; NICU, neonatal intensive care unit. Values are presented as mean or %. *p*-values calculated using the Kruskal–Wallis test for continuous variables and chi-square test for categorical variables.

Gestational age at delivery was similar across groups (~38–39 weeks). Preterm birth rates showed no significant difference (*p* = 0.89), with values ranging from 13.6% to 15.9%.

LBW was most common in the ≥60-month group (16.6%), approaching statistical significance (*p* = 0.09).

NICU admission was highest in the 6–11-month group (29.4%), with borderline significance (*p* = 0.05).

Apgar scores did not significantly differ (Table 2).

### 3.3. Quartile and Tertile Analyses

Quartile-based analysis revealed that Q4 (≥59.9 months) had higher early preterm birth and LBW rates than Q1. Tertile analysis produced similar findings, with T3 exhibiting the highest maternal age and moderately increased LBW/NICU rates (Table 4 and Table 5).

### 3.4. Influence of Previous Cesarean Delivery

Women with a prior cesarean delivery were older and had higher gravidity/parity. Preterm birth (12.1% vs. 10.8%, *p* = 0.03) and NICU admission (23.9% vs. 18.6%, *p* = 0.02) were significantly more common among those with previous cesarean delivery (Table 6).

### 3.5. Statistical Comparison and Regression Models

Bivariate analysis showed significant differences in maternal age (*p* < 0.001) and gravidity (*p* < 0.001) across classical IPI groups, while LBW approached significance (*p* = 0.05) and NICU did not reach significance (*p* = 0.23).

### 3.6. Regression for Preterm Birth

After adjustment, intervals of 24–59 months (aOR 0.48, *p* = 0.002) and ≥60 months (aOR 0.58, *p* = 0.042) were independently protective against preterm birth.

- Maternal age increased preterm birth risk by 7% per year (OR 1.07; *p* = 0.001) (Table 7). The discrepancy between the raw preterm birth rates (highest in the ≥60 month group, as seen in Table 3) and the results of the multivariable model is primarily explained by the strong confounding effect of maternal age. While the crude OR (0.87; 95% CI 0.55–1.38) suggested a slight increase in risk, this was driven by the significantly higher maternal age in the long-IPI group (mean 33.5 years).

**Table 7:** Logistic regression analysis for neonatal outcomes (reference: 12–23 months).

Variable/IPI Category	Crude OR (95% CI)	Adjusted OR * (95% CI)	*p*-Value (Adj)
IPI < 6 months	0.22 (0.05–0.94)	0.28 (0.06–1.24)	0.095
IPI 6–11 months	0.59 (0.28–1.21)	0.67 (0.32–1.40)	0.290
IPI 24–59 months	0.56 (0.35–0.88)	0.48 (0.29–0.77)	0.002
IPI ≥ 60 months	0.87 (0.55–1.38)	0.58 (0.35–0.98)	0.042
Maternal age (per year)	1.09 (1.05–1.13)	1.07 (1.03–1.12)	0.001

Abbreviations: OR, odds ratio; CI, confidence interval; IPI, interpregnancy interval. * Adjusted for maternal age, gravidity, and previous cesarean delivery.

Regression for NICU Admission

- IPI 6–11 months was associated with almost two-fold increased NICU risk (OR 2.29; *p* = 0.002).
- Previous cesarean delivery independently increased NICU admission (OR 1.35; *p* = 0.048).
- Long IPI (24–59 months) showed a nonsignificant upward trend (*p* = 0.073).

(Table 8) Intervals ≥ 60 months also increased NICU risk (aOR 1.61, *p* = 0.036).

- In the short-IPI groups (<12 months), the majority of NICU admissions (approx. 65%) were attributed to respiratory distress and transient tachypnea of the newborn, particularly among infants delivered near-term.

**Table 8:** Logistic regression for NICU admission (reference: 12–23 months).

Variable/IPI Category	Crude OR (95% CI)	Adjusted OR * (95% CI)	*p*-Value (Adj)
IPI < 6 months	0.97 (0.44–2.16)	1.10 (0.47–2.52)	0.820
IPI 6–11 months	2.14 (1.26–3.62)	2.29 (1.33–3.94)	0.002
IPI 24–59 months	1.38 (0.93–2.05)	1.44 (0.96–2.15)	0.073
IPI ≥ 60 months	1.66 (1.09–2.54)	1.61 (1.02–2.54)	0.036
Previous cesarean	1.38 (1.01–1.88)	1.35 (1.01–1.85)	0.048

Abbreviations: OR, odds ratio; CI, confidence interval; IPI, interpregnancy interval; * Adjusted for maternal age, gravidity, and previous cesarean delivery.

To further investigate the impact of IPI on low birth weight (LBW), a dedicated logistic regression model was constructed (Table 9). While raw data indicated a higher prevalence of LBW in the ≥60-month group (16.6% vs. 0.0–11.9%), in other groups, the multivariable analysis showed that this association did not reach conventional statistical significance after adjusting for maternal age, gravidity, and previous cesarean delivery (adjusted OR, 1.28; 95% CI, 0.88–1.86; *p* = 0.198). For LBW, the observed trend in the ≥60-month group was not statistically significant after adjustment (aOR, 1.33; *p* = 0.279), suggesting the effect is primarily age-mediated. This finding suggests that while a trend toward increased LBW exists in long IPI, it is not as robust an independent predictor as it is for preterm birth or NICU admission in this cohort.

## 4. Summary

Both short IPI (6–11 months) and very long IPI (≥60 months) were independently associated with increased NICU admission. While crude data suggested a higher prevalence of LBW in intervals exceeding 24 months, this association was largely driven by the confounding effect of maternal age. Maternal age remained a robust independent predictor of preterm birth, and previous cesarean delivery significantly increased neonatal morbidity.

## 5. Discussion

This study evaluated neonatal outcomes using a refined delivery-to-conception interval and demonstrated that risk patterns are not linear across the IPI spectrum. A key strength of our disaggregated analysis was the identification of a risk “U-curve.” While short IPIs (6–11 months) were consistently linked to higher NICU admission (29.4%), our findings for long intervals revealed a significant confounding effect of maternal age [12,13].

Once maternal age was statistically isolated, intervals exceeding 24 months (24–59 and ≥60 months) emerged as independently protective against preterm birth (aOR 0.48 and 0.58, respectively), challenging the raw data trends that suggested higher risks in older cohorts [10].

Maternal age emerged as a robust independent predictor of preterm birth, consistent with previous large-cohort analyses [14]. Advanced maternal age is known to affect placental function, vascular integrity, and inflammatory pathways, all contributing to earlier delivery. The observed association between previous cesarean delivery and elevated NICU admission parallels published evidence linking cesarean birth with increased neonatal respiratory morbidity [15]. This consistency suggests that the observed associations between maternal age, interpregnancy interval, and neonatal outcomes are likely to reflect broader biological and demographic patterns rather than population-specific effects.

Several biological mechanisms may explain the increased neonatal morbidity associated with short interpregnancy intervals. Maternal nutritional depletion, particularly reduced folate and iron stores, has been proposed as one of the principal pathways linking short birth spacing with adverse pregnancy outcomes. When pregnancies occur in rapid succession, maternal physiological reserves may not have sufficient time to recover, which can impair placental development and fetal growth. In addition, incomplete endometrial recovery and persistent uterine or cervical inflammation following the previous pregnancy may disrupt normal implantation and vascular remodeling. These biological processes may lead to suboptimal placental function, thereby increasing the risk of fetal growth restriction, preterm birth, and subsequent neonatal complications. These mechanisms contribute to premature labor, fetal growth restriction, and increased neonatal morbidity [16,17].

The observed increase in NICU admission (aOR 1.61) and LBW prevalence (16.6%) in the ≥60-month group supports the “physiological regression” hypothesis. This theory suggests that the maternal body eventually loses the favorable vascular and immunological adaptations gained from a prior pregnancy, returning to a “primiparous-like” state. This regression, coupled with the natural accumulation of chronic maternal conditions over time and reduced vascular memory response, explains why neonatal morbidity rises again after five years of spacing. This combination increases the risk for LBW and NICU admission, even when the risk of preterm birth is reduced after age adjustment.

In contrast, very long interpregnancy intervals may increase neonatal risk through different biological pathways. One widely discussed hypothesis is the concept of “physiological regression,” which suggests that the maternal body gradually loses the beneficial vascular and metabolic adaptations acquired during a previous pregnancy. As the interval between pregnancies increases, these physiological adaptations may revert to a state similar to that of a first pregnancy. Furthermore, longer intervals are frequently associated with increasing maternal age and the potential accumulation of chronic maternal conditions, both of which may adversely affect placental function and fetal development. Reduced vascular and immunological memory responses may therefore contribute to the increased rates of low birth weight and NICU admission observed in very long intervals [18,19].

The use of three complementary classification strategies—classical cut-offs, quartiles, and tertiles—provides a robust validation of our findings. Across all methods, consistent trends were observed, particularly regarding the increased neonatal morbidity in short intervals and the emergence of risk gradients in longer intervals. Crucially, while the classical ≥24-month category is broad and may mask risk gradients, our quartile analysis (Q4 ≥ 60.3 months) provided a nuanced understanding by revealing that the highest risks for LBW and NICU admission are concentrated in very long intervals, whereas Q3 (33.0–60.2 months) represented a potential window of reduced risk for preterm birth.

This study has several notable strengths. First, the relatively large cohort of 1194 participants provides sufficient statistical power to detect clinically meaningful associations between interpregnancy interval and neonatal outcomes. Second, the use of three complementary classification strategies—classical cut-offs, quartiles, and tertiles—allowed a more comprehensive evaluation of potential threshold effects across the IPI spectrum. Third, the study incorporated multiple clinically relevant neonatal outcomes, including preterm birth, low birth weight, and NICU admission, thereby offering a broad perspective on neonatal morbidity. Finally, the use of multivariable regression models adjusting for biologically plausible confounders strengthens the robustness and interpretability of the findings.

## 6. Limitations

This study also has several limitations that should be considered when interpreting the findings. First, the retrospective design limits the ability to establish causal relationships between interpregnancy interval and neonatal outcomes. Second, the study was conducted at a single tertiary referral center in Istanbul, which may limit the generalizability of the results to other populations with different demographic characteristics, healthcare systems, or obstetric practices. Third, information on several potentially important confounding variables, including socioeconomic status, pregnancy intention, and maternal nutritional status, was not available in the dataset. Because socioeconomic conditions may influence both reproductive behavior and neonatal health, residual confounding cannot be completely excluded. Finally, the relatively small number of women in the <6-month IPI category may have reduced statistical precision for this subgroup and widened confidence intervals.

A major strength of this study is the resolution of systematic measurement bias by utilizing delivery-to-conception intervals and the application of three complementary classification systems to validate risk thresholds. Unlike previous studies that relied on delivery-to-LMP estimates, our corrected methodology provides a more accurate reflection of maternal physiological recovery. However, certain limitations remain. The retrospective design limits causal inference, and data on socioeconomic status and pregnancy intention were unavailable. Furthermore, only 4.2% (n = 50) of our cohort fell into the <6-month IPI category, which limits the statistical precision for this specific subgroup.

Although previous studies have suggested that an interpregnancy interval of approximately 18–24 months may be associated with improved perinatal outcomes, the present study contributes to the literature by applying three complementary classification models within the same cohort. This multiclassification approach enabled a direct comparison of competing neonatal risks across different interval thresholds and demonstrated consistent patterns of increased NICU admission in both short and very long intervals. By integrating classical cut-offs with distribution-based analyses, our study provides a more nuanced understanding of risk gradients across the IPI spectrum and offers clinically relevant insights for postpartum birth-spacing counseling.

## 7. Clinical Implications

These findings reinforce the necessity of individualized postpartum counseling focused on optimal birth spacing to mitigate adverse neonatal outcomes. While our multivariable analysis indicates that intervals exceeding 24 months are independently protective against preterm birth after adjusting for maternal age, a window of 18–24 months remains the most clinically balanced recommendation. This target optimizes the trade-off between the increased NICU admission risks associated with short IPIs (<12 months) and the rising trends in NICU admission and LBW observed in very long intervals (≥60 months). Integrating IPI assessment and reproductive planning into routine postpartum care is particularly vital for high-risk populations—including young mothers, women of advanced maternal age, and those with a history of previous cesarean delivery—to effectively reduce neonatal morbidity and prevent unintended rapid repeat pregnancies.

Our findings reflect the complex reproductive landscape of a high-volume tertiary center in Istanbul. The observed prevalence of short IPIs may be partly attributed to cultural norms and limited postpartum counseling in busy clinical settings, whereas long IPIs (≥60 months) increasingly reflect the ‘delayed childbearing’ trend seen in urban Turkish populations. These sociocultural factors do not just determine the interval itself; they also influence the maternal nutritional baseline and the presence of chronic comorbidities, which we found to be significant mediators of neonatal risk in long intervals.

Future studies incorporating prospective designs and detailed socioeconomic and behavioral data are needed to further clarify the complex determinants and consequences of interpregnancy intervals.

## 8. Conclusions

This study underscores that both short and excessively long interpregnancy intervals are associated with distinct neonatal risks. Short IPI (6–11 months) and very long IPI (≥60 months) were identified as independent risk factors for NICU admission. While intervals exceeding 24 months demonstrated a significant protective effect against preterm birth after adjusting for the confounding impact of maternal age, the rising trend toward LBW and NICU admission in intervals beyond 5 years suggests that birth-spacing recommendations should be nuanced. The 18–24-month window remains the most clinically prudent target to mitigate the combined risks of acute maternal depletion associated with short spacing and the physiological regression associated with very long spacing. Individualized birth-spacing guidance is essential to optimize neonatal health outcomes.

## Figures and Tables

**Table 1:** Baseline maternal characteristics according to interpregnancy interval categories.

Variable	<6 Months (*n* = 52)	6–11 Months (*n* = 114)	12–23 Months (*n* = 264)	≥24 Months (*n* = 768)	*p*-Value
Maternal age (years, mean ± SD)	24.7 ± 5.0	26.5 ± 4.6	27.7 ± 4.7	31.5 ± 5.0	<0.001
Gravidity (mean ± SD)	2.2 ± 0.4	2.5 ± 1.0	2.7 ± 1.3	2.8 ± 1.0	<0.001
Previous cesarean delivery (%)	38.5%	49.1%	51.5%	55.7%	0.04

Abbreviations: Values are presented as mean ± standard deviation or percentage. Continuous variables were compared using the Kruskal–Wallis test and categorical variables using the chi-square test.

**Table 2:** Distribution of patients across classical IPI categories (*N* = 1194).

IPI	Number of Patients (*n*)	Percentage (%)
<6 months	50	4.2%
6–11 months	102	8.5%
12–23 months (reference)	270	22.6%
24–59 months	470	39.4%
≥60 months	302	25.3%
Total	1194	100.0%

Abbreviations: IPI, interpregnancy interval.

**Table 4:** Maternal and neonatal findings by quartile IPI groups.

IPI Quartile	Range (Months)	Maternal Age (Years, Mean)	Preterm Birth (%)	LBW (%)	NICU Admission (%)
Q1	≤17.8	26.7	12.7%	5.3%	20.7%
Q2	17.9–32.9	28.7	12.8%	14.8%	23.5%
Q3	33.0–60.2	30.7	7.4%	9.4%	16.8%
Q4	≥60.3	33.6	14.1%	16.8%	24.8%

Abbreviations: IPI, interpregnancy interval; LBW, low birth weight; NICU, neonatal intensive care unit. Values are presented as mean or %. *p*-values calculated using the Kruskal–Wallis test (continuous variables) and chi-square test (categorical variables).

**Table 5:** Maternal and neonatal findings by tertile IPI groups.

IPI Tertile	Range (Months)	Maternal Age (Years, Mean)	Preterm Birth (%)	LBW (%)	NICU Admission (%)
T1	≤22.7	26.9	13.1%	9.5%	19.1%
T2	22.8–48.6	29.6	10.6%	10.6%	22.6%
T3	≥48.7	33.2	11.6%	14.6%	22.6%

Abbreviations: IPI, interpregnancy interval; LBW, low birth weight; NICU, neonatal intensive care unit. Values are presented as mean or %. *p*-values calculated using Kruskal–Wallis or chi-square test as appropriate.

**Table 6:** Comparison according to previous cesarean history.

Group	Maternal Age (Years, Mean)	Preterm Birth (%)	LBW (%)	NICU Admission (%)
Previous Cesarean (+)	30.8	12.1%	11.1%	23.9%
No Previous Cesarean (−)	28.9	10.8%	11.5%	18.6%

Abbreviations: LBW, low birth weight; NICU, neonatal intensive care unit. Values are presented as mean or %. *p*-values calculated using independent-samples *t* test or chi-square test.

**Table 9:** Logistic regression model for low birth weight (LBW, <2500 g).

Variable/IPI Category	Crude OR (95% CI)	Adjusted OR * (95% CI)	*p*-Value (Adj)
IPI ≥ 60 months	1.47 (0.91–2.38)	1.33 (0.78–2.26)	0.279

Abbreviations: OR, odds ratio; CI, confidence interval; IPI, interpregnancy interval. * Adjusted for maternal age, gravidity, and previous cesarean delivery.

## Data Availability

The data underlying this study are available from the corresponding author upon reasonable request. Due to institutional privacy policies and protection of patient information, the dataset cannot be publicly shared but can be provided in de-identified form to qualified researchers.
